# Patient Safety Framework in Healthcare Regulatory System of Nepal: A Call for Action

**DOI:** 10.31729/jnma.9160

**Published:** 2025-07-31

**Authors:** Satish Kumar Deo, Srijana Shakya, Sujaya Gupta

**Affiliations:** 1 Nepal Medical Council, Bansbari, Kathmandu, Nepal; 2 Department of Clinical Pharmacology, Maharajgunj Medical Campus, Institute of Medicine, Maharajgunj, Kathmandu, Nepal; 3 Department of Periodontics and Oral Implantology, Dental Programme, Kathmandu Medical College, Duwakot, Bhaktapur, Nepal.

**Keywords:** *adverse events*, *healthcare*, *patient safety*, *regulatory system*

## Abstract

Patient safety in Nepal’s healthcare system remains a critical challenge due to fragmented regulatory frameworks, resource constraints, and inadequate monitoring mechanisms. Despite multiple regulatory bodies overseeing healthcare services, their lack of coordination limits effectiveness. Learning from international models, Nepal can enhance patient safety by establishing a unified governance system, strengthened monitoring mechanisms, and increased budget allocations. A dedicated patient safety framework, including stakeholder collaboration, capacity-building, and a national reporting system, is crucial. Increasing healthcare funding to at least 5% of Gross Domestic Product (GDP) would demonstrate Nepal’s commitment towards improving patient safety and achieving global healthcare standards.

## INTRODUCTION

Healthcare regulatory compliance involves adherence to laws and guidelines that ensure patient rights, data protection, fraud prevention, and high-quality care.^1^ To improve patient safety, regulatory bodies establish policies, conduct inspections, provide accreditations, and oversee professional development.^2^ Despite these efforts, fragmented regulations limit efficiency, highlighting need for robust framework. Annually, one in ten patients experience harm, and unsafe practices contribute over three million deaths. Additionally, unsafe care consumes up to 12.6% of healthcare budgets in high-income nations, costing approximately $878 billion.^3,4^ Nepal faces similar challenges, necessitating coordinated patient safety measures. This article explores a structured framework for Nepal, integrating global strategies to enhance patient safety.

## GLOBAL AND REGIONAL SCENARIO OF PATIENT SAFETY REGULATIONS

Different countries have taken different approaches to establish a Patient Safety Culture in their respective countries which align with the recommendations of World Health Organization (WHO).^4^

Canada, Australia, and United States of America (USA) are concerned for patient safety which led to the establishment of the Canadian Patient Safety Institute, the Australian Council for Safety and Quality in Health Care, and the Agency for Healthcare Research and Quality respectively.^5,6^ Canada has been able to conduct research to study various aspects of adverse events, make collaborative efforts with stakeholders to implement mechanisms that ensure patient safety and operate reporting system for monitoring patient safety incidents. As a result, resources to educate health professionals, home-providers, and policy makers were published, leading to decline in patient safety incidents and introduction of new methods to measure hospital harm.^7-9^ Whereas, Australian government has developed the National Safety and Quality Health Service (NSQHS) Standards, including patient safety emphasizing in governance for safety and quality, prevention and control of Health Care Associated Infections (HCAI), medication safety, clinical handover, responding to acute deterioration and prevention of falls etc. Consequently, the number of accredited hospitals that ensures patient safety has been increased, the rate of patient safety incidents has declined, there is better documentation of adverse events, and decrease in admission at Intensive Care Unit and in-hospital cardiac arrest.^7,8^ Similarly, USA has enforced Safety Improvement Act 2005 which authorizes to create Patient Safety Organizations and develop common formats for uniform reporting of Patient Safety events.^9^ Sweden has enforced the Patient Safety Act (2010:659), a holistic legislation characterized by comprehensive laws that emphasize systemic, proactive approaches to healthcare, prioritizing patient safety and harm prevention.^4^

The Government of India formed a multi-stakeholder Patient Safety Expert Group in 2016 for developing a National Patient Safety Implementation Framework (NPSIF). The aim of NPSIF is to address the fragmented and overlapping regulatory arrangements implemented by various stakeholders across the country.^10^ Similarly, Philippines has developed national policy for efficient implementation and institutionalization of the patient safety programme in health facilities.^4^ Additionally, Thailand has introduced Patient Safety Policy, also referred to as the 3P safety policy, which includes health professionals as well as general public.^4^ The Ministry of Health in Cambodia is drafting the Law on Administration of Health Services to further improve healthcare quality through detailed regulations and the establishment of a National Accreditation System, to be overseen by an independent public institution.^11^

**Table 1:** List of service regulators and their scope of functions.^14,17^

Regulators of Services	Scope of Functions
1. Curative Service Division (CSD) - Ministry of Health and Population (MOHP)	Assess impact of health programs on the population. Monitor programs via government and non-government committees. Develop and implement monitoring and evaluation policies. Conduct studies and research on health services. Facilitate monthly, quarterly, and annual reviews at the provincial level. Setting Minimum Service Standards.
2. Quality Standard and Regulation Section (QSRS) - MOHP	Sets standards of health services, drugs, and infrastructures. Monitoring and evaluation of quality health services.
3. Medical Education Commission	Accreditation of medical education institution. Common entrance exam. Monitoring and evaluation of quality of medical education. Setting policy related to medical education.
4. Department of Drug Administration	Accreditation of pharmacies and pharmaceutical companies. Sets policies, standard and guidelines related to medicine and pharmacy operation. Monitors Adverse Drug Reaction (ADR) monitoring system. National pharmacovigilance center.

**Table 2:** Professional regulators and their functions.^18-21^

Regulators of Professionals	Scope of Functions
1. Nepal Medical Council	Takes actions against adverse events reported by public. Licensing exams and registration. Sets Code of Conduct for doctors. Sets policies related to registration and health services. Sets Good Clinical Practices guidelines. Conducts Continuing Professional Development (CPD) trainings for medical professionals. Sets other relevant clinical guidelines and protocols. Accreditation of Fellowship programs. Accreditation of CPD activities conducted by various institutions.
2. Nepal Nursing Council	Licensing exams and registration. Sets policies related to registration. Sets Code of Conduct for nurses.
3. Nepal Health Professional Council	Licensing exams and registration. Sets policies related to registration. Sets Code of Conduct for health professionals.
4. Nepal Pharmacy Council	Licensing exams and registration. Sets policies related to registration. Sets Code of Conduct for pharmacists.

## SCENARIO OF PATIENT SAFETY IN NEPAL

In Nepal, patient safety faces humongous challenges. Medical professionals are often overburdened, caring for far more patients than those in developed countries, and have limited access to modern equipment, especially in remote areas. According to WHO’s recommendation, for every 10,000 people, there should be 45 healthcare professionals available, including physicians, nurses, and midwives. Whereas, in Nepal only 34 health professionals are available for every 10,000 persons. This increases the risk of errors.

The shortage of skilled professionals, combined with insufficient training, further compromises care quality. Additionally, poor communication among healthcare providers and with patients leads to medical errors. Addressing these issues through enforcement of regulatory policies, professional trainings, standardized protocols, and increased awareness of patient safety is essential to improving outcomes.^12,13^

## REGULATORY SYSTEM OF HEALTH SECTOR IN NEPAL

The regulatory bodies of health sector of Nepal oversee organizations, services, professionals, and healthcare products through various monitoring mechanisms. It also sets policies, develops quality standards, offer accreditation services, and support professionals through education and training (Table 1, 2).

## SUMMARY

The healthcare system in Nepal faces significant challenges due to limited resources, inadequate training, and a fragmented monitoring framework for adverse events. The current regulatory setup, involving multiple bodies such as the Nepal Medical Council, Nursing Council, Health Professional Council, and Pharmacy Council, struggles with disorganizations that compromise patient safety. Overburdened professionals, nonuniform protocols, and communication gaps further worsen risks.

Insufficient financial commitment with only 4.6% of the total budget hinders effective implementation of patient safety measures.^23^ By taking lessons from countries like Canada, Australia, and USA, Nepal can benefit from centralized regulatory body for patient safety with dedicated budgets, and policy development to initiate improvements in quality care.

## CONCLUSIONS

A unified, multi-stakeholder expert group is essential to reorganize patient safety initiatives. The expert group should comprise healthcare and medical education experts, government representatives, patient advocates, and institutional leaders to design and implement unified policies. Strengthening capacity-building, particularly in rural regions, alongside establishing a national level central reporting system for adverse events, can foster transparency and accountability. Increasing the healthcare budget to at least 5% of Gross Domestic Product (GDP) would further align Nepal with global patient safety standards, indicating a commitment to improving health outcomes through integrated governance and resource allocation.22
